# Complete Surgical Excision Is Necessary following Vacuum-Assisted Biopsy for Breast Cancer

**DOI:** 10.3390/curroncol29120734

**Published:** 2022-11-30

**Authors:** Jung Ho Park, So Eun Ahn, Sanghwa Kim, Mi Jung Kwon, Yong Joon Suh, Doyil Kim

**Affiliations:** 1 Division of Breast and Endocrine Surgery, Hallym University Sacred Heart Hospital, Anyang 14068, Republic of Korea; 2 Department of Pathology, Hallym University Sacred Heart Hospital, Anyang 14068, Republic of Korea

**Keywords:** breast neoplasms, surgery, ultrasonography, interventional

## Abstract

Vacuum-assisted breast biopsy (VABB) has been replacing excisional biopsy in the treatment of benign breast lesions. Complete surgical excision is still needed for the lesions occasionally diagnosed with breast cancer after VABB. We aimed to characterize residual tumors after VABB and define a subset of patients who do not need surgical excision after VABB. From a retrospective database, we identified patients diagnosed with breast cancer after VABB guided with ultrasonography. Patients who underwent stereotactic biopsies were excluded. We reviewed clinicopathologic data and radiologic findings of the sample. We identified 48 patients with 49 lesions. After surgical excision, the residual tumors were identified in 40 (81.6%) lesions, and there was no residual tumor in nine (18.3%) patients. Imaging studies could not accurately locate residual tumors after VABB. A small tumor size on a VABB specimen was associated with no residual tumor on final pathology. However, residual tumors were identified in four (40%) of 10 lesions with a pathologic tumor size less than 0.5 cm. In conclusion, complete surgical excision remains the primary option for most of the patients diagnosed with breast cancer after VABB. Imaging surveillance without surgery should be carefully applied for selected low-risk patients.

## 1. Introduction

Surgical treatment for breast cancer can cause scars, deformities, and lymphedema, all of which negatively affect quality of life [1]. With the development of systemic treatment, modern surgical methods have evolved to decrease the extent of surgery and improve cosmetic outcomes [2]. Conventional mastectomy has largely been replaced by nipple-sparing mastectomy with breast reconstruction. The resection margin of the breast-conserving surgery has been minimized. Complete axillary lymph node dissection has been replaced with targeted axillary dissection following neoadjuvant chemotherapy.

Vacuum-assisted breast biopsy (VABB) is a minimally invasive method for acquiring biopsy specimens. Compared to core-needle biopsy, VABB acquires a significantly larger amount of tissue and minimizes radiologic–pathologic discordance. VABB has been widely adopted as a substitute for surgical resection for benign breast conditions [3]. Although guidelines recommend open surgical excision for high-risk lesions [4], many clinicians favor surveillance for various reasons. Given the excellent cosmetic outcome, the indications of VABB are being extended [5,6]. Clinical trials underway to omit surgery in a subset of patients who respond well to neoadjuvant chemotherapy [7].

When breast cancer is identified after VABB, complete surgical excision is routinely performed to achieve adequate resection margins. However, it is unknown whether breast cancer can be solely treated with VABB. For achieving satisfactory local control, the residual tumor burden should be sufficiently low so that it can be controlled with radiation treatment [8]. Our aim was to describe the characteristics of residual tumors after VABB and identify a subset of patients who do not require complete surgical excision after VABB.

## 2. Materials and Methods

### 2.1. Patient Identification

From a retrospective database, we reviewed the electronic medical records of 3289 patients with breast cancer who had underwent surgery between 2003 and 2021 at a single tertiary institution (Supplementary Figure S1). Then, we identified patients who underwent VABB and subsequent surgical excision for the same lesion. We included patients diagnosed with ductal carcinoma in situ (DCIS) or invasive breast cancer after VABB guided by ultrasonography. We excluded the patients who underwent tomosynthesis-guided stereotactic biopsy. We excluded patients who were diagnosed with lobular carcinoma in situ or only benign tumor. Demographic, radiologic, and pathologic data were collected.

**Figure 1:**
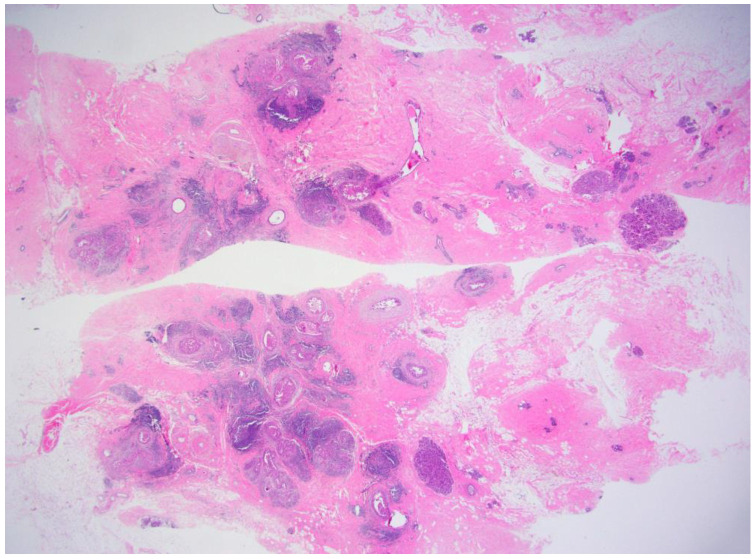
Microscopic findings of the breast cancer identified on VABB. There were multiple foci of microinvasive carcinoma (H&E, ×30). Resection margin was deemed involved because of the fragmented specimen. There was no residual tumor after complete surgical excision.

**Figure 2:**
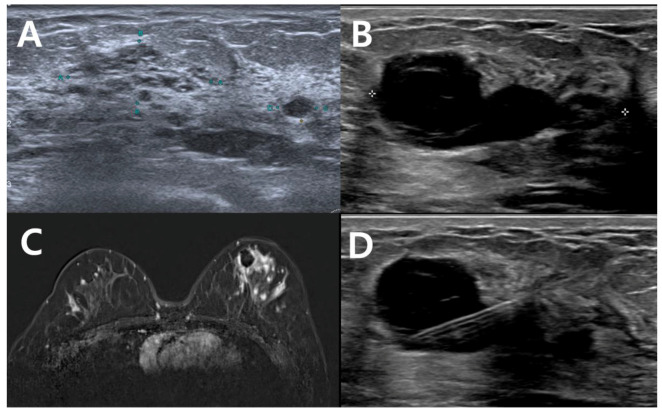
Radiologic findings of a representative case. (**A**) The initial non-mass lesion was excised by VABB and diagnosed with DCIS. (**B**) Second-look ultrasonography did not reveal residual tumor. (**C**) MRI revealed residual non-mass lesion surrounding post-VABB cavity. (**D**) The post-VABB site was localized with metallic wire and excised widely. Final pathology was a 4.2 cm sized DCIS.

Most of the patients underwent VABB at the primary clinics and were subsequently diagnosed with breast cancer. Pathologic slides from the initial procedure were reviewed by institutional pathologists (Figure 1). Patients were evaluated using ultrasonography and magnetic resonance imaging (MRI) to find residual tumors (Figure 2A–C). When residual tumors were suspected, the lesions were localized with hook-wires and excised during surgery (Figure 2C). The post-VABB hematoma was widely excided with adequate margins. The sentinel lymph node biopsy was performed at the surgeon’s discretion. The extent of the remnant tumors was described in the pathology report.

### 2.2. Statistical Analysis

Continuous variables were demonstrated as mean and standard deviation. Categorical variables were presented as frequencies and percentages. Categorical variables were compared between groups using a Chi-square test. Continuous variables were compared between groups using a Student’s t test. The threshold for the *p*-value was set at 0.05. Statistical analyses were performed using the Statistical Package for Social Science (version 24.0; IBM corp, Armonk, NY, USA).

## 3. Results

### 3.1. Clincopathological Characteristics of the Study Patients

A total of 52 patients were referred by 21 surgeons from 18 clinics. After slide review, three patients were diagnosed with benign pathology and excluded from analysis. We identified 48 patients with 49 lesions. After surgical excision, residual tumors were identified on 40 (81.6%) lesions, and there were no residual tumors on 9 (18.4%). The clinicopathological characteristics of the study patients were summarized in Table 1. There was no significant difference on the two groups. Regarding the initial biopsy method, 7 (14.3%) lesions underwent fine-needle aspiration, 16 (32.7%) lesions underwent core-needle biopsy, and 26 (53.1%) lesions underwent VABB.

### 3.2. Diagnostic Performances of Imaging Modalities for Detecting Remnant Lesions

Patients were evaluated with imaging modalities after VABB before surgery. Among the 49 lesions, 26 (53.1%) underwent mammography, 47 (95.9%) underwent ultrasonography, and 49 (100%) underwent MRI. The accuracies of the mammography, ultrasonography, and MRI were 30.8%, 59.6%, and 61.2%, respectively (Table 2). The MRI detected an additional five lesions of which ultrasonographic findings were negative (Figure 2C). However, three lesions detected on ultrasonography were false-negative on MRI.

Overall, imaging modalities did not accurately localize residual tumors. Even for the 11 (22.0%) lesions, which were negative for all three radiologic tests, eight (72.7%) lesions had residual tumors after surgery.

### 3.3. Sentinel Lymph Node Biopsy

All except one patient underwent a sentinel lymph node biopsy. Only one (2.0%) patient was positive for sentinel lymph node biopsy. Among the seven lymph nodes excised, one sentinel lymph node was positive and six non-sentinel lymph nodes were negative. Subsequent axillary dissection was performed, and an additional three metastatic lymph nodes were identified. Two (4.1%) patients had isolated tumor cells, and two (4.1%) patients had micrometases on the permanent pathology of sentinel lymph nodes, all of which were negative on frozen biopsy.

### 3.4. The Association of Tumor Size on VABB Specimen and Remnant Tumor on Pathology

The median size of the residual tumor was 1.4 cm (range, 0.2–4.2 cm). No histological upgrade of the breast lesion was observed after the surgical excision. The only variable associated with a residual tumor was the pathologic tumor size on the VABB specimen (Table 3). The pathologic tumor size on the VABB specimen was available for 27 (55.1%) lesions. The patients with no residual tumor on final pathology showed smaller tumor size on the VABB specimen than the patients with residual tumors (0.38 cm vs. 0.89 cm, *p* < 0.01). Among 10 patients with a tumor size less than or equal to 0.5 cm, six (60%) patients did not show residual tumor after surgery. When the tumor size on the VABB specimen was larger than 0.5 cm, residual tumor existed for all lesions.

## 4. Discussion

We found a high proportion of residual tumors present after VABB, and surgical excision was necessary to clear the residual tumor. The proportion of residual tumors after VABB for breast cancer was 81.6%. This high proportion of residual tumors is consistent with previous studies using mammography-guided stereotactic biopsy, which reported that the residual tumor was identified in 62.7–85.7% of the patient sample [9,10,11,12,13,14,15]. The performance of VABB was inferior to that of incomplete surgical excision, of which a residual tumor was identified in 34.5% after re-excision [16]. The median size of the residual tumor was 1.4 cm, which is comparable to that of clinically positive disease. Even for tumors less than 0.5 cm, four (40%) of ten lesions had residual tumors on excision. Complete surgical excision was needed to achieve clear resection margin, regardless of the tumor size.

The disappearance of the lesion on ultrasound after VABB did not guarantee complete pathological removal. The diagnostic performance of imaging studies is lower than expected, and this finding is consistent with a previous study [9]. After excluding patients who were negative on the three imaging modalities, residual tumors were identified in 7 (70.0%) of the 10 patients. Mammography showed a high specificity for identifying residual malignant microcalcifications after VABB. However, microscopic residual tumors may not be identifiable with mammography [11,17]. There exists an additional benefit of MRI, although MRI immediately after surgery is complicated by cicatrical changes [18]. As imaging modalities do not accurately localize the residual tumor, surgical excision is more extensive because the entire biopsy cavity should be excised with adequate margin.

This limitation of VABB is due to the inherent mechanism of the VABB device. The role of VABB is primarily focused on tissue acquisition. The probe vacuums the mass into the notch, and the blade fragments the lesion lying in the notch. This concept contradicts conventional en bloc resection of the tumor and makes it difficult to achieve a clear resection margin. Although the actual risk of tumor seeding after VABB is trivial [19], a theoretic risk exists. Ablation techniques using thermal energy can complement VABB. In a study that reported 11 cases treated with VABB combined with laser ablation, 90% of the treated patients did not show residual tumors after surgical excision [20]. In a study using a novel breast lesion excision system that combined radiofrequency with VABB, complete excision was possible for 95.8% of subcentimeter breast cancers [21].

Another weakness of the minimally invasive method is that the axillary nodal status cannot be evaluated. While sentinel lymph node biopsy is unnecessary for patients who are diagnosed with DCIS on VABB [22], we advocate for the use of both sentinel lymph node biopsy and frozen sections when invasive cancer is identified. However, recent clinical trials have focused on omitting sentinel lymph node biopsy for early breast cancer with a clinically negative axilla [23]. Undertreatment and axillary recurrence may occur because axillary ultrasonography does not completely exclude advanced nodal disease [24]. One of our patients with clinically negative axilla underwent sentinel lymph node biopsy; one lymph node was positive and six non-sentinel lymph nodes were negative. Subsequently, complete axillary nodal clearance was performed. Three additional metastatic lymph nodes were identified and she was upstaged to N2.

Despite the limitations mentioned above, VABB has been actively studied for the treatment of low grade DCIS. Surgery with or without radiation is traditionally recommended for the treatment of DCIS. However, conventional surgical excision has been challenged by the issue of overtreatment [25]. A large proportion of DCIS remains indolent for a long time and can be identified only by screening. Adequate local control does not lead to overall survival gain. Three prospective trials are ongoing for the active surveillance of low-grade DCIS [26,27,28]. The results of the trials will confirm the safety of the active surveillance approach. Another recent trial required stricter criteria and only recruited unifocal DCIS with low-grade and strong ER positivity [29,30]. Because it takes a long time to obtain the results of these prospective trials, the active surveillance of low-grade DCIS should be carefully recommended. The treatment for individual patients should be carefully planned considering the tumor extent, histologic grade, receptor status, and patient preference.

Our study has several limitations. First, the small sample size of our study restricts the statistical power and generalizability. We could not identify a specific subset of patients who do not require surgical excision after VABB. Second, due to the retrospective design, we could not observe clinical outcomes without complete surgical excision. Incomplete surgical excision does not always result in recurrence. Prospective studies may demonstrate the actual clinical benefits of surgical excision in the future. Third, our study was prone to referral bias because most patients were referred from the primary clinics. Each referring physician applies different indications and protocols for VABB, uses different devices and equipment, and has varying degrees of experiences. Atypical ductal hyperplasia is often not referred for surgery, and radiologic surveillance is performed in the primary clinic. Nevertheless, our data included heterogeneous population comparable to that of a multicenter study.

## 5. Conclusions

Lumpectomy or mastectomy with axillary nodal surgery remains the standard treatment for breast cancer after VABB. Without surgery, the oncologic outcomes of VABB for breast cancer would be inconsistent and unreliable. The disappearance of the lesion on imaging did not guarantee the complete removal of the tumor. The management of breast cancer diagnosis after VABB can be individualized based on tumor characteristics and patient preferences.

## Figures and Tables

**Table 1:** Comparison between patients with or without residual tumor after complete surgical excision.

	No Residual Tumor (n = 9)	Residual Tumor on Excision (n = 40)	*p*
Age (years)	49.44 ± 17.01	51.20 ± 10.67	0.773
Menopausal status			1.000
Premenopausal	4 (44.4%)	19 (47.5%)	
Postmenopausal	5 (55.6%)	21 (52.5%)	
BMI (kg/m^2^)	24.18 ± 4.22	23.45 ± 3.23	0.631
Tumor size on US *	1.42 ± 0.79	1.54 ± 0.80	0.698
BI-RADS category ^†^			0.781
3	1 (33.3%)	6 (54.5%)	
4A	1 (33.3%)	3 (27.3%)	
4B	1 (33.3%)	2 (18.2%)	
Initial biopsy method			0.725
FNA	2 (22.2%)	5 (12.5%)	
CNB	3 (33.3%)	13 (32.5%)	
Upfront VABB	4 (44.4%)	22 (55.0%)	
Surgery			0.569
BCS	9 (100%)	35 (87.5%)	
TM	0	5 (12.5%)	
Histology			0.128
DCIS	2 (22.2%)	20 (50.0%)	
Microinvasive carcinoma	2 (22.2%)	2 (5.0%)	
IDC	5 (55.6%)	18 (45.0%)	
HR status			0.364
Negative	3 (33.3%)	7 (17.5%)	
Positive	6 (66.7%)	33 (82.5%)	
HER2 status ^‡^			0.326
Negative	7 (77.8%)	20 (51.3%)	
Equivocal	1 (11.1%)	13 (33.3%)	
Positive	1 (11.1%)	6 (15.4%)	

* available for 45 lesions; ^†^ available for 14 lesions; ^‡^ available for 48 lesions. BMI, body-mass index; US, ultrasonography; BI-RADS, breast imaging reporting and data system; FNA, fine needle aspiration; CNB, core needle biopsy; BCS, breast-conserving surgery; TM, total mastectomy; DCIS, ductal carcinoma in situ; IDC, invasive ductal carcinoma; HR, hormonal receptor; HER2, human epidermal growth factor receptor2.

**Table 2:** Diagnostic performances of the imaging modalities for identifying residual tumors after VABB.

	Mammography (n = 26)	Ultrasonography (n = 47)	MRI (n = 49)
Sensitivity	5/23 (21.7%)	22/38 (57.9%)	24/40 (60.0%)
Specificity	3/3 (100%)	6/9 (66.7%)	6/9 (66.7%)
PPV	5/5 (100%)	22/25 (88.0%)	24/27 (88.9%)
NPV	3/22 (13.6%)	6/22 (27.2%)	6/22 (27.2%)
Accuracy	8/26 (30.8%)	28/47 (59.6%)	30/49 (61.2%)

MRI, magnetic resonance imaging; PPV, positive predictive value; NPV, negative predictive value.

**Table 3:** Association between tumor size and residual lesion.

Pathologic Tumor Size	No Residual Tumor (n = 9)	Residual Tumor on Excision (n = 40)
≤0.5 cm	6	4
0.6~1 cm	0	12
1.1~2 cm	0	5
No data	3	19

## Data Availability

The data presented in this study are available on request from the corresponding author. The data are not publicly available due to the risk of personal information extrusion.

## Supplementary Materials

The following supporting information can be downloaded at: https://www.mdpi.com/article/10.3390/curroncol29120734/s1, Figure S1: Flow diagram of the study design.
