# Supplementary material for: Complete Surgical Excision Is Necessary following Vacuum-Assisted Biopsy for Breast Cancer
Source: Curr Oncol. 2022 Nov 30;29(12):9357–64. doi: 10.3390/curroncol29120734 (PMC9777068; doi:10.3390/curroncol29120734)
Supplement: Supplementary file 1 [file curroncol-29-00734-s001.zip › curroncol-2054887-supplementary.pdf]

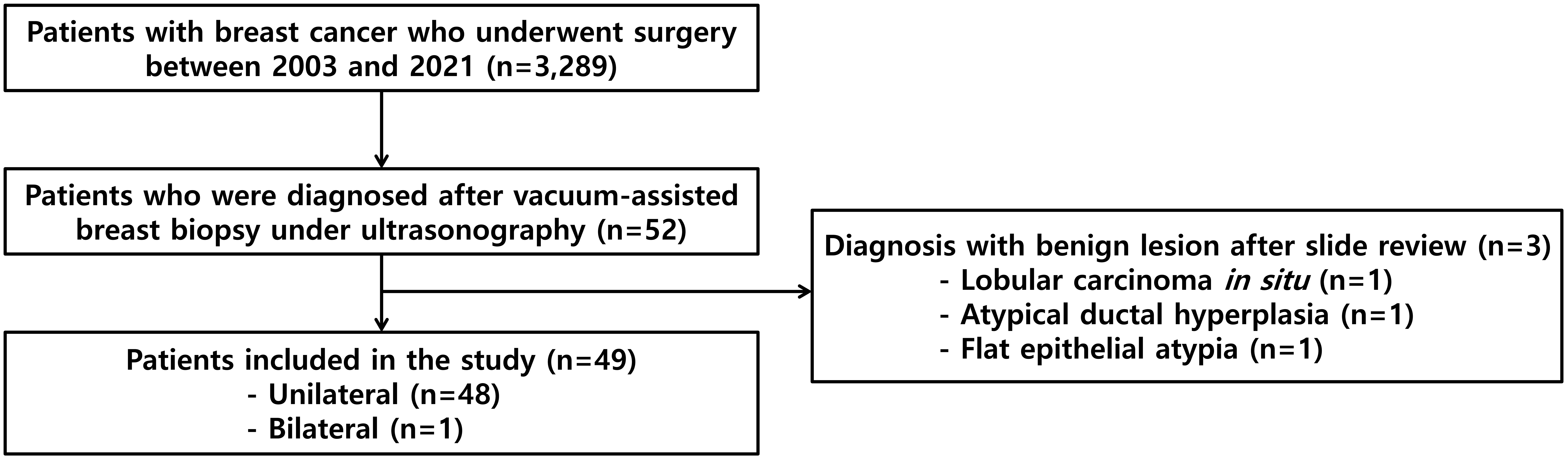
Patients with breast cancer who underwent surgery between 2003 and 2021 (n=3,289)
Patients who were diagnosed after vacuum-assisted breast biopsy under ultrasonography (n=52)
Diagnosis with benign lesion after slide review (n=3)
- Lobular carcinoma in situ (n=1)
- Atypical ductal hyperplasia (n=1)
- Flat epithelial atypia (n=1)
Patients included in the study (n=49)
- Unilateral (n=48)
- Bilateral (n=1)
